# A Synergic Strategy: Adipose-Derived Stem Cell Spheroids Seeded on 3D-Printed PLA/CHA Scaffolds Implanted in a Bone Critical-Size Defect Model

**DOI:** 10.3390/jfb14120555

**Published:** 2023-11-21

**Authors:** Gabriela S. Kronemberger, Thiago Nunes Palhares, Alexandre Malta Rossi, Brunno R. F. Verçosa, Suelen C. Sartoretto, Rodrigo Resende, Marcelo J. Uzeda, Adriana T. N. N. Alves, Gutemberg G. Alves, Mônica D. Calasans-Maia, José Mauro Granjeiro, Leandra Santos Baptista

**Affiliations:** 1 Nucleus of Multidisciplinary Research in Biology (Numpex-Bio), Federal University of Rio de Janeiro (UFRJ) Xerém, Duque de Caxias 25245-390, RJ, Brazil; soareskg@tcd.ie (G.S.K.); brunno.vercoza@gmail.com (B.R.F.V.); 2 Laboratory of Eukariotic Cells, National Institute of Metrology, Quality and Technology (Inmetro), Duque de Caxias 25250-020, RJ, Brazil; 3 Post-Graduation Program of Translational Biomedicine (Biotrans), Unigranrio, Campus I, Duque de Caxias 25071-202, RJ, Brazil; 4 Brazilian Center for Physics Research, Xavier Sigaud 150, Urca 22290-180, RJ, Brazil; thiagonup@gmail.com (T.N.P.); rossi@cbpf.br (A.M.R.); 5 Laboratory of Clinical Research in Odontology, Fluminense Federal University (UFF), Niterói 24020-140, RJ, Brazil; susartoretto@hotmail.com (S.C.S.); resende.r@hotmail.com (R.R.); mjuzeda@gmail.com (M.J.U.); aterezinhanovellino@gmail.com (A.T.N.N.A.); gutopepe@yahoo.com.br (G.G.A.); monicacalasansmaia@gmail.com (M.D.C.-M.)

**Keywords:** bone, osteogenesis, critical-size defects, tissue engineering, spheroids, synergic strategy, scaffolds, 3D printing, bone regeneration

## Abstract

Bone critical-size defects and non-union fractures have no intrinsic capacity for self-healing. In this context, the emergence of bone engineering has allowed the development of functional alternatives. The aim of this study was to evaluate the capacity of ASC spheroids in bone regeneration using a synergic strategy with 3D-printed scaffolds made from poly (lactic acid) (PLA) and nanostructured hydroxyapatite doped with carbonate ions (CHA) in a rat model of cranial critical-size defect. In summary, a set of results suggests that ASC spheroidal constructs promoted bone regeneration. In vitro results showed that ASC spheroids were able to spread and interact with the 3D-printed scaffold, synthesizing crucial growth factors and cytokines for bone regeneration, such as VEGF. Histological results after 3 and 6 months of implantation showed the formation of new bone tissue in the PLA/CHA scaffolds that were seeded with ASC spheroids. In conclusion, the presence of ASC spheroids in the PLA/CHA 3D-printed scaffolds seems to successfully promote bone formation, which can be crucial for a significant clinical improvement in critical bone defect regeneration.

## 1. Introduction

Bone tissue has an excellent capacity for self-healing from non-critical-size defects [1]. However, most cases of bone critical-size defects and non-union fractures caused by trauma, tumor resection, infection, or congenital malformations will lead to a clinical intervention [2]. The gold standard treatment for these defects is autologous bone graft [3]. However, this treatment has some drawbacks, such as donor site morbidity, which is considered the biggest challenge. Allogenic bone grafts have garnered attention in the clinic [4]. However, they are devitalized prior to implantation, and the process starts with the loss of osteoinductive potential together with immunogenicity issues.

“*Scaffold-free*” techniques are being largely explored in tissue engineering. In this approach, cells interact directly with one another through a process called “*self-assembly*”, which leads to the formation of spheroids [5]. The main advantages related to the use of spheroids are their ability to provide superior cellular heterogeneity, nutrient and oxygen gradients, extracellular matrix deposition, and tissue-like gene expression profiles [6]. Spheroids produced from bone marrow mesenchymal stem cells (bmMSCs) and adipose-derived stem/stromal cells (ASCs) have been already applied as successful developmental models to recapitulate osteogenesis in vitro [7,8,9]. They are capable of fusing with other spheroids, which allows them to be used as building blocks for bone tissue engineering [10,11]. Although bmMSCs and ASC spheroids have great potential to form bones, few regenerative pre-clinical studies have been performed to achieve this goal throughout the years [7,12,13].

Suenaga and collaborators (2015) [7] fabricated osteogenically induced bmMSC spheroids, combining them with β-tricalcium phosphate (β-TCP) granules for their implantation into rat calvaria critical-size bone defect. The results showed a higher proportion of new bone formation in the group administered osteogenically induced bmMSC spheroids. Lee and collaborators (2020) [12] delivered ASC spheroids, formed by 3D-printed microchamber system, into rat calvaria critical-size bone defects. The microchamber was coated with platelet-derived growth factors (PDGF) and morphogenetic protein 2 (BMP-2). The main results showed that this group was able to form a larger area of new bone in these defects, compared with the control groups. Findeisen and collaborators (2021) [13] produced bmMSC spheroids and implanted them into rat femoral defects and compared them with a cell suspension. The results showed that the spheroid group produced a significantly higher density of bone mineral.

As discussed, few studies have been published with ASC spheroids that regenerate bone tissues through the years. Gurumurthy and collaborators (2017) [14] showed that ASC spheroids had a higher osteogenic potential when compared to traditional monolayer techniques. The ASC spheroids also showed a high viability after the culture period. Ahmad and collaborators (2018) [15] developed composite ASC spheroids combined with polymeric fibers. Their main results showed that the constructs formed a mineralized functional tissue. Yamada and collaborators (2022) [16] implanted ASC spheroids in a rat calvaria defect. They showed a significant bone regeneration in the group containing the spheroids when compared to the control group. 

One innovative approach that is being explored in tissue engineering consists of a synergic strategy that combines spheroids and scaffolds to achieve better biomechanical properties, higher cell density, and extracellular matrix production, resulting in an improvement in functionality of the final construct [17]. In this context, nanostructured hydroxyapatite scaffolds are being highly explored for bone tissue engineering applications due to their high osteoinductive potential [18,19]. These scaffolds can also be 3D printed using an extrusion-based technique to develop more versatile and custom-sized scaffolds that will fit better into a patient’s bone defect, thereby improving the bone regeneration process [20,21]. 

So far, to the best of our knowledge, only two studies have applied this synergic approach to regenerate bone tissue in vivo [7,12]. Therefore, the aim of this study was to evaluate the capacity of ASC spheroids seeded on the surface of poly (lactic acid) (PLA) and nanostructured hydroxyapatite doped with carbonate ions (CHA) on 3D-printed scaffolds to regenerate bone critical-size defects in rats.

## 2. Material and Methods

### 2.1. Monolayer 2D Culture of Human Adipose Stem/Stromal Cells (ASCs)

ASCs were isolated from healthy human donors (aged 18–55 years old) and cryopreserved as previously described [22]. The isolation procedure of the human ASCs was performed according to the Research Ethics Committee of Clementino Fraga Filho University Hospital, Federal University of Rio de Janeiro, Brazil (25818719.4.0000.5257). Next, the ASCs were seeded into 75 cm^2^ flasks and maintained in culture as previously described [10,11]. For their growth, the ASC cell suspension at passage one was thawed and seeded into 175 cm^2^ flasks and maintained in chemically defined TheraPEAK™ MSCGM-CD™, Mesenchymal Stem Cell Medium (Lonza, São Paulo, Brazil), at 37 °C in a humid atmosphere with 5% carbon dioxide (CO_2_). After passage three, the cells were used for spheroid fabrication. For all the experiments performed in this study, we have used one donor and three independent experiments for each assay.

### 2.2. Spheroid 3D Culture

The ASC spheroids were fabricated in a micro-molded non-adhesive hydrogel system. The micro-molded non-adhesive hydrogel wells were fabricated using a solution of 2% (*w*/*v*) Ultrapure Agarose (Invitrogen, São Paulo, Brazil) in a 0.9% NaCl solution, formed from silicone molds containing 81 resections (MicroTissues^®^ 3D Petri Dish^®^, Sigma Aldrich, St. Louis, MO, USA). Next, a total cell suspension of 2 × 10^6^ ASCs at passage three was gently seeded into the micro-molded non-adhesive hydrogels. After 40 min of the seeding, the culture media was added in each well. The culture media was made using low glucose Dulbecco’s modified eagle medium (DMEM) supplemented with Insulin-Transferrin-Selenium (ITS) (1×; Sigma), 1.25 μg/mL human albumin (Farma Biagini SPA, Rio de Janeiro, Brazil), 50 μg/mL ascorbic acid (Sigma), 100 μg/mL penicillin, and 100 μg/mL streptomycin (Sigma). The ASC spheroids were then maintained for 2 days in the micro-molded non-adhesive hydrogels at 37 °C in a humid atmosphere with 5% carbon dioxide (CO_2_) and 21% oxygen (O_2_). 

### 2.3. Spheroid Diameter Measurements

After 24 h of ASC spheroid formation and up to 1 week after the culture, a total of 15 images were acquired using a phase contrast microscope (Primo Vert, Zeiss, Pittsburgh, Pennsylvania, USA) equipped with a digital camera. The width and length measurements of each spheroid were determined using the AxioVision Rel. 4.6 software (Zeiss, Jena, Germany). Next, the diameter ratio of each spheroid was obtained by dividing width by length to obtain the spheroid sphericity. These analyses were performed using one cell donor and three independent experiments using fifteen spheroids from each sample.

### 2.4. 3D Printing of PLA/CHA Scaffolds

The 3D printing process for the fabrication of the PLA/CHA scaffolds has been described previously in detail [23]. Briefly, the 3D printing filaments of the Filament Deposition Modeling (FDM) printing technique were produced in a twin-screw extruder printer (Haake Rheomex OS Prw16, Thermo Scientific, Waltham, MA, USA). For the preparation of the composite, the PLA pellets were manually mixed according to the desired proportion (% by mass) of 10% CHA. Next, these pellets were added to the extruder feed hopper, where they were mixed using a melting temperature profile of 150/160/170/180/185/190 °C for the 3D printing process. At the extruder outlet, a circular matrix was used in order to achieve a filament shape for the 3D printing process.

### 2.5. Seeding of ASC Spheroids on the Surface of 3D-Printed PLA/CHA Scaffolds

**Figure 1:**
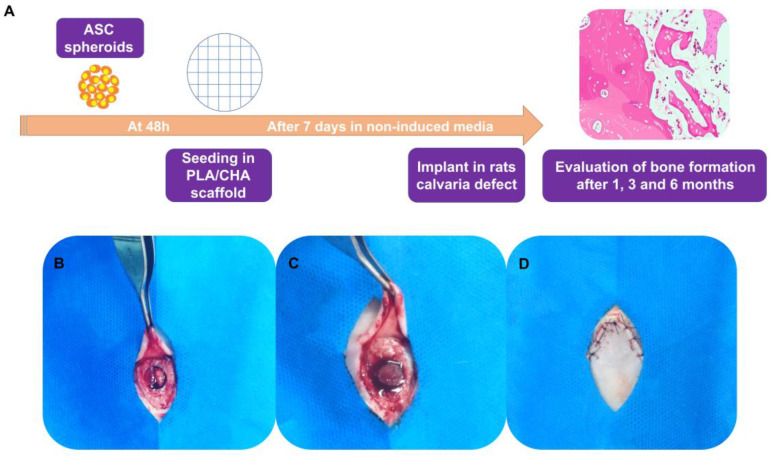
Graphical abstract and surgical procedure of constructs implanted in 8 mm calvaria defects in rats. (**A**) ASC spheroids were fabricated using non-adherent micromolded agarose hydrogel technique. After 48 h, spheroids were seeded on the surface of PLA/CHA scaffolds. The constructs (PLA/CHA scaffolds + ASC spheroids) were maintained for 1 week in culture media and then implanted in calvaria defects of rats. The evaluation of new bone formation was conducted by histological analysis after 1, 3, and 6 months of the implantation period. (**B**) Surgical bone defect exposure. (**C**) ASC spheroidal constructs implanted in situ. (**D**) Defect sutured. ASC: adipose-derived stem/stromal cell; PLA/CHA: poly (lactic acid) and nanostructured hydroxyapatite doped with carbonate ions.

Initially, the 3D-printed PLA/CHA scaffolds were placed in wells of a 48-well plate. Next, a total of 162 ASC spheroids, which corresponds to a total density of 4 × 10^6^ cells, were gradually dispersed at once on the surface of 3D-printed PLA/CHA scaffolds in a total volume of 200 μL. The manual dispersion of spheroids was performed in all areas of the 3D-printed scaffolds. Next, the 3D-printed scaffolds containing the seeded ASC spheroids were maintained in a humid atmosphere at 37 °C, at 5% CO_2_ and 21% CO_2_ for 24 h, in a final volume of 100 µL of culture media of the spheroids. The low volume of media was used in order to achieve a better adhesion of the ASC spheroids on the surfaces of the 3D-printed PLA/CHA scaffolds. After 24 h, 500 µL of the culture media for spheroids was slowly added in each well, and the constructs (ASC spheroids + PLA/CHA scaffolds) were maintained at 37 °C, at 5% CO_2_ and 21% CO_2_. Finally, the constructs were maintained for a total period of 7 days in culture for in vitro analyses and for the surgical procedures (Figure 1A).

### 2.6. Scanning Electron Transmission (SEM) Analysis

In order to evaluate the interaction of the ASC spheroids on the surface of the 3D-printed PLA/CHA scaffolds, SEM analyses were performed. Initially, the constructs were washed three times with 0.01 M PBS for 5 min and then fixed in a solution of 2.5% glutaraldehyde (Sigma) in 0.1 M sodium cacodylate buffer (Sigma) for 2 h in the dark. Next, the samples were washed twice with sodium cacodylate buffer for 5 min and post-fixed in osmium tetroxide (Sigma) 1% diluted in 0.1 M sodium cacodylate and kept for 40 min in the dark. Sequentially, the samples were washed 3 times for 5 min with 0.1 M sodium cacodylate buffer. After that period, samples were dehydrated by serial washes in ethanol solutions for 10 min each, in concentrations of 30%, 50%, 70%, 90%, and finally 100% ethanol (three times). All steps were performed at room temperature. After dehydration, the samples were slowly dried in a critical point device (Leica/CPDO30, São Paulo, Brazil) and afterwards covered with 10 nm gold layer by the sputtering equipment (DENTON VACUUM, Leica, São Paulo, Brazil).

In addition, the energy-dispersive X-ray spectroscopy (EDS) analyses were performed for the PLA/CHA scaffolds to identify the presence of calcium (Ca) and phosphorus in the samples. For EDS analyses, the detector used was the Oxford X-MaxN 20 mm^2^.

### 2.7. Analysis of Multiple Secreted Proteins

At week 1 of ASC spheroidal constructs culture, the medium was changed to fresh medium, and the samples were maintained in culture for 24 h. In addition, a scaffold without spheroids seeded was maintained in culture for the same period as a control condition. After that period, the total volume of supernatant was collected in Eppendorfs and immediately frozen and stored at −80 °C. In order to quantify the proteins secreted in the supernatant, a Luminex xMAP technology based on a magnetic bead panel was used for recognition of the following mediators: human MIP-1β, IFN-y, interleukin-1ra (IL-1ra), IL-5, GM-CSF, TNFα, RANTES, IL-2, IL-1β, Eotaxin, bFGF, VEGF, PDGF-BB, IP-10, IL-13, IL-4, IL-5, MCP-1, IL-8, MIP-1a, IL-10, G-CSF, IL-15, IL-7, IL-12p70, IL-17ra, and IL-9 (27-plex panel, Bio Rad Laboratories Inc., Hercules, CA, USA). The quantification was performed using the Bio-Plex Magpix apparatus (Bio Rad Laboratories Inc., Hercules, CA, USA), following the manufacturer’s instructions. Next, the concentration of each secreted product was quantified using the xPONENT v3.1 software (LuminexCorp^®^, Austin, TX, USA), and the results were expressed as picograms per milliliter (ρg/mL). The control group (scaffold without seeded spheroids) values were used to normalize the results of the ASC spheroids seeded in the PLA/CHA group. The assay was performed from one cell donor and three independent experiments using five replicates from each sample. 

### 2.8. In Vivo Study

This study was approved by the Ethics Committee on the Use of Animals at the Universidade Federal Fluminense (CEUA-UFF) (approval number: 1474030618). In this study, 72 female Wistar rats between two and four months old, weighing between 180 and 300 g, were provided by the Laboratory Animal Nucleus (NAL, Niteroi, Rj, Brazil). All surgical procedures on the animals were performed at the Animal Experimentation Laboratory (LEA, Niteroi, RJ, Brazil). The animals were kept during the entire period of this study in mini-isolators (*n* = 3) and fed with pelleted feed and water at will. The animals were randomly distributed using envelopes for their allocation to each experimental group (*n* = 6), totaling 24 animals for each experimental period (1, 3, and 6 months).

For the surgeries, the animals were deprived of solid food six hours before the procedures and then were administered general anesthesia. Anesthetic induction and maintenance were performed with the administration of 100 mg/kg of Ketamine (Francotar^®^—Virbac—Jurubatura, SP, Brazil), 10 mg/kg of Xylazine (Sedazine^®^—Fort Dodge, Iowa, USA), and 5 mg/kg of Midazolam (Eurofarma), which were administered intramuscularly. After observing the absence of pain reflexes, trichotomy was performed in the animal’s skullcap region using a sharp razor blade (Enox platinum^®^, Enox, São Paulo, SP, Brazil), and the region was cleaned with 2% chlorhexidine soap (Riohex 2%^®^, Rio de Janeiro, Rj, Brazil). Then, the animals were placed in the ventral decubitus position, and a semilunar incision of 2 cm length was made with a surgical blade, number 15C (Solidor^®^, Tampa, Florida, USA), followed by a periosteal mucus detachment of the region using Molt #9 (Molt Quinelato^®^, Tampa, Florida, USA) and the bone exposure of the entire skull of the animal. The bone defect was performed with a trephine bur with an 8 mm internal diameter (Harte group^®^, Doylestown, PA, USA) (Figure 1B–D). 

Subsequently, the division of the implants was carried out, and the animals were allocated into three experimental groups for this study: (1) PLA/CHA; (2) PLA/CHA seeded with ASC spheroids; and (3) Clot. After the end of the surgical procedures, the tissues were repositioned by suturing with a 5.0 Nylon thread (Mononylon^®^, Cidade Monções, SP, Brazil). Subsequently, the animals received Meloxicam (Maxicam^®^, Croydon, United Kingdom) injection 15 mg/1.5 mL, 1 mg/kg every 24 h, and Tramadol Hydrochloride (Tramal^®^, Milwaukee, WI, USA), 10 mg/kg, subcutaneously every 8 h for three days. The animals were euthanized at 1, 3, and 6 months post-surgical procedures by applying a lethal dose of general anesthetic (Thiopental, 150 mg/kg).

### 2.9. Histological Processing

For the ASC spheroid samples, at the end of 7 days of culture, the spheroids were initially harvested and washed twice using 0.01 M PBS (Sigma). Next, samples were fixed in 4% paraformaldehyde (Sigma) solution for 1 h. Sequentially, ASC spheroids were washed twice with 0.01 M PBS solution and dehydrated with serial washes in ethanol (Sigma) for 20 min each, in concentrations of 70%, 90%, and 100%. Later, samples were washed twice in xylene (Sigma) for 20 min and embedded in wax. The wax blocks were cut with a microtome (Slee Medical, Cut 5062, Nieder-Olm, Germany) and stained with Hematoxylin (Sigma) and Eosin (Sigma) solutions.

The calvaria bone block samples containing the constructs were collected with a long carbide spherical drill #6 (Low Spherical #6 FG^®^, São Paulo, SP, Brazil), coupled with a micromotor (Marathon 3 Champion Micromotor – Talma, São Paulo, SP, Brazil). Next, the samples from each experimental group were dissected to remove the soft tissue and fixed for 48 h in 4% formalin solution (pH 7.4) and subjected to the standard histological procedure. Briefly, samples were washed for 1 h in running water, demineralized in decalcifier solution (Allkimia^®^—Campinas, São Paulo, SP, Brazil) for 48 h, again washed in running water, dehydrated in increasing concentrations of ethanol (70, 80, 90, and 100%, Rialcool^®^, Rioquímica, Brazil) for 1 h each, cleared in two xylene baths (Xylene^®^, Ehningen, Germany) of 1 h each, and finally embedded in paraffin. Furthermore, these samples were cut with a microtome (Jung-Leica RM 2054) into slices of 5 μm thickness and stained with Hematoxylin (Sigma) and Eosin (Sigma) and Masson’s Trichrome (Sigma) for the descriptive evaluation of newly formed bone and connective tissue. For the descriptive histological analysis, a Light Field Light Microscope (OLYMPUS^®^, Tokyo, Japan) was used. Images were obtained with an optical microscope-coupled camera (OLYMPUS^®^ SC100, Tokio, Japan), associated with the CELLSENS^®^1.9 DigitalImage software (Tokio, Japan).

### 2.10. Statistical Analysis

Non-parametric Mann–Whitney *t*-test was utilized for the comparison between 0 h and week 1 ASC spheroid diameter values. For the in vitro analysis of multiple secreted proteins, the comparisons of growth factors, interleukins, and chemokines secreted by the ASC spheroids at week 1 of culture were performed with a *one-way* ANOVA test. The results in the graphs are expressed as mean ± standard error. Differences were considered statistically significant when *p* < 0.05. Statistical analyses were performed using GraphPad Prism 6.0 software (GraphPad Inc., La Jolla, CA, USA).

## 3. Results

### 3.1. ASC Spheroids Are Homogeneous in Size

**Figure 2:**
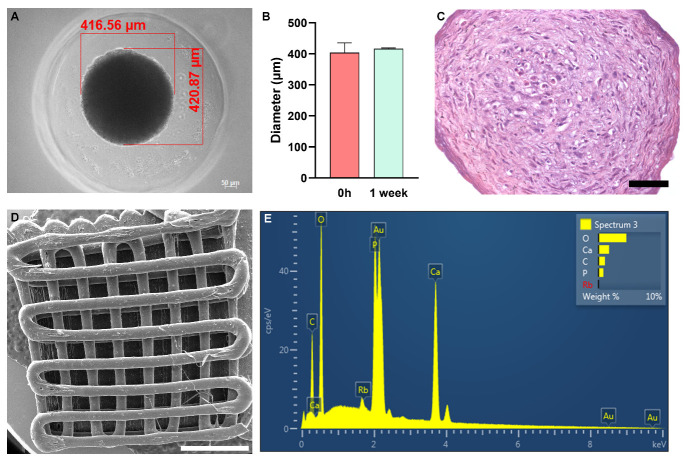
ASC spheroids are homogeneous in size, and 3D-printed PLA/CHA scaffolds contain calcium (Ca) and phosphorus (P) in their surface. (**A**) Representative image of one ASC spheroid inside one agarose resection. The red lines represent the measurements of width and length. (**B**) Diameter ratio of ASC spheroids after 0 h and 1 week of culture. (**C**) Hematoxylin and Eosin staining of one ASC spheroid after 1 week of culture. Notably, a rounded cell morphology at the center and a fibroblastic morphology at the periphery of spheroids are observed. (**D**) Scanning electron microscopy (SEM) image of one 3D-printed PLA/CHA scaffold. (**E**) Energy-dispersive X-ray spectroscopy (EDS) analysis of the PLA/CHA scaffolds showing the presence of Ca and P element signals originating from their surface. ASC: adipose-derived stem/stromal cell; and PLA/CHA: poly (lactic acid) and nanostructured hydroxyapatite doped with carbonate ions. Scale bars: (**C**): 50 µm; and (**D**): 2 mm.

After 7 days of culture, it was observed that ASC spheroids had an average diameter of 400 µm (Figure 2A,B). The Hematoxylin and Eosin staining showed a rounded cell morphology at the center of the spheroid and a fibroblastic morphology at its periphery after 7 days of culture (Figure 2C). 

The PLA/CHA scaffolds have homogeneous filaments and porous sizes (Figure 2D). The EDS analysis showed the presence of Ca and P in the surface of the scaffold (Figure 2E), confirming the efficiency of CHA incorporation. 

### 3.2. ASCs Are Released from Spheroids Spread on Most Areas of the PLA/CHA Scaffolds

**Figure 3:**
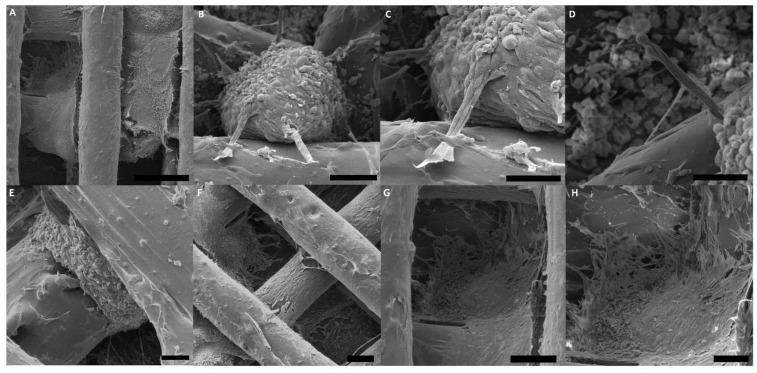
ASCs derived from spheroids were able to spread on the surface of the PLA/CHA scaffolds. (**A**–**H**) ASC spheroids seeded on the surface of PLA/CHA scaffold at week 1. (**A**) ASC spheroids fused and spread on different areas of the PLA/CHA scaffold. (**B**–**D**) ASCs derived from spheroids were able to migrate and adhere to the surface of the PLA/CHA scaffold. (**E**) ASC spheroids fused to interact with the filaments of the PLA/CHA scaffold. (**F**) Cells from the ASC spheroids were able to interact and adhere inside the pores of the PLA/CHA scaffold. (**G**,**H**) The cells showed mostly a fibroblastic morphology when they made contact with the PLA/CHA scaffold. Scale bars: (**A**)—500 µm; (**B**,**C**)—50 µm; (**D**)—20 µm; (**E**,**F**)—200 µm; (**G**)—100 µm; and (**H**)—50 µm. ASC: adipose-derived stem/stromal cell; PLA/CHA: poly (lactic acid) and nanostructured hydroxyapatite doped with carbonate ions.

Initially, the interaction of ASC spheroids with PLA/CHA scaffolds in vitro was evaluated using scanning electron microscope (SEM) analysis (Figure 3). After being seeded on the surface of the PLA/CHA scaffold, the ASC spheroids showed a high capacity of adhesion in all areas of the PLA/CHA scaffold (Figure 3A,B). In addition, ASCs derived from spheroids were able to interact with the surface of the PLA/CHA scaffolds (Figure 3C–H). These migrating cells mostly showed a fibroblastic morphology after adhering to the surface of the PLA/CHA scaffold (Figure 3F–H). 

### 3.3. ASC Spheroidal Constructs Show a Low Secretion of Pro-Inflammatory Mediators and a High Secretion of VEGF In Vitro

**Figure 4:**
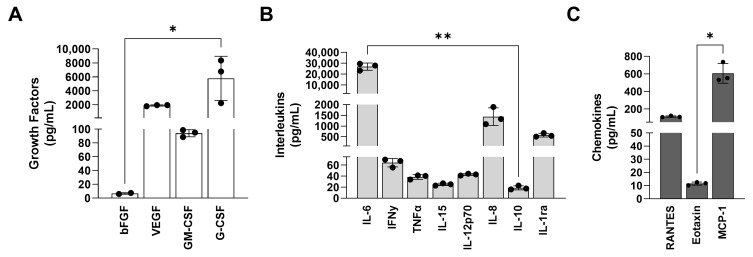
ASC spheroid constructs showed a high secretion of VEGF at week 1 of culture. Quantification of soluble mediators secreted by ASC spheroids seeded on PLA/CHA scaffold at week 1 of culture. (**A**) Growth factors. (**B**) Interleukins. (**C**) Chemokines. The data are expressed as mean  ±  SD. The asterisks indicate *p*-values obtained by non-paired *one-way* ANOVA followed by Kruskal–Wallis multiple comparisons (* *p* < 0.05; ** *p* < 0.001). ASC: adipose-derived stem/stromal cells; PLA/CHA: poly (lactic acid) and nanostructured hydroxyapatite doped with carbonate ions; IL: interleukin; IL-6: interleukin-6; IL-8: interleukin-8; IL-10: interleukin-10; IL-12p70: interleukin-12; IL-15: interleukin-15; IFN-y: interferon-γ; MCP-1: monocyte chemoattractant protein-1; VEGF: vascular endothelial growth factor; GM-CSF: granulocyte-macrophage colony-stimulating factor; and G-CSF: granulocyte colony-stimulating factor.

The levels of the pro-inflammatory cytokines, i.e., GM-CSF (Figure 4A), IFNy (Figure 4B), IL-15 (Figure 4B), and IL-12p70 (Figure 4B), were more than 10 times reduced in PLA/CHA scaffolds seeded with ASC spheroids at week 1 of in vitro culture when compared to the levels of VEGF (Figure 4A) and IL-8 (Figure 4B), which are well known to promote angiogenesis, and IL-6 (Figure 4B) that plays a crucial role during osteogenesis. 

### 3.4. ASC Spheroidal Constructs Were Able to Produce New Bone Tissue at the Center of Critical-Size Defects

**Figure 5:**
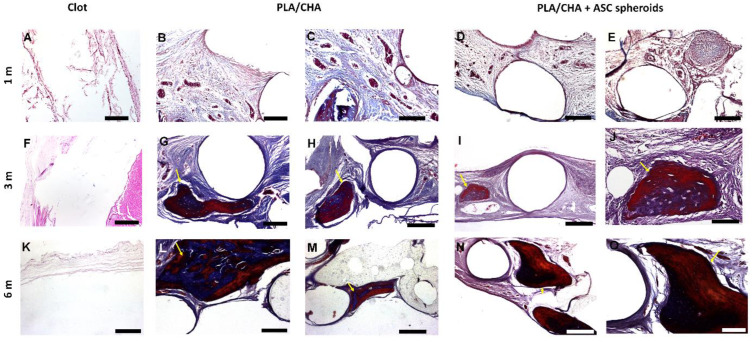
Histological analysis of calvaria defects after 1, 3, and 6 months of implantation. (**A**,**F**,**G**) Masson’s Trichrome staining of Clot group after 1, 3, and 6 months. (**B**,**C**,**G**,**H**,**L**,**M**) Masson’s Trichrome staining of PLA/CHA group after 1, 3, and 6 months, respectively. (**D**,**E**,**I**,**J**,**N**,**O**) Masson’s Trichrome staining of with ASC spheroids construct after 1, 3 and 6 months, respectively. The new bone tissue formed at month 3 in PLA/CHA ((**G**,**H**), arrow) and PLA/CHA seeded with ASC spheroidal constructs groups ((**I**,**J**), arrow). The new bone tissue formed at month 6 in PLA/CHA ((**L**,**M**), arrow) and PLA/CHA seeded with ASC spheroidal constructs groups ((**N**,**O**), arrow). Results are representative of 6 rats/groups. Scale bars: (**A**)—200 µm; (**B**)—200 µm; (**C**)—100 µm; (**D**)—200 µm; (**E**)—200 µm; (**F**)—200 µm; (**G**)—200 µm; (**H**)—200 µm; (**I**)—200 µm; (**J**)—50 µm; (**K**)—200 µm; (**L**)—200 µm; (**M**)—200 µm; (**N**)—200 µm; and (**O**)—100 µm. ASC: adipose-derived stem/stromal cell; PLA/CHA: poly (lactic acid) and nanostructured hydroxyapatite doped with carbonate ions.

The biological response and new bone formation between the implanted groups were evaluated at months 1, 3, and 6 using histological analysis (Figure 5). Initially, we observed that none of the scaffolds lost their structural integrity, and no significant degradation was found after the in vivo assay. In addition, no inflammatory response was observed throughout the analyzed period by any of the implanted groups.

At month 1, in the PLA/CHA (Figure 5B,C) and in the PLA/CHA seeded with ASC spheroids groups (Figure 5D,E), no bone tissue was formed. In addition, in both groups, we observed connective tissue in close contact with areas of virtual biomaterial (Figure 5B–E). At month 3, in the PLA/CHA (Figure 5G,H) and in the PLA/CHA seeded with ASC spheroids groups (Figure 5I,J), it was possible to observe areas of new bone tissue in close contact with areas of virtual biomaterial, compared with month 1. At month 6 (Figure 5L–O), both groups presented a mature bone tissue formation. In the Clot groups at 1, 3, and 6 months, it was not possible to observe the formation of bone tissue (Figure 5A,F,K).

## 4. Discussion

In this study, ASC spheroids seeded on the surface of 3D-printed PLA/CHA scaffolds showed a high potential to promote bone regeneration in a rat calvaria critical-size defect model. Our ASC spheroidal constructs showed an active secretion of cytokines and growth factors when cultured in vitro, together with new bone tissue formed in the central area of the calvarial defect. To the best of our knowledge, this is the first study based on the synergic strategy (scaffold-free combined with scaffold-based) to promote bone regeneration using ASC spheroids combined with a 3D-printed PLA/CHA scaffold. 

Initially, ASC spheroids were able to adhere and spread onto the PLA/CHA scaffold. In the past, several studies explored the seeding of MSCs on the surface of polymeric scaffolds for bone tissue engineering applications [12,24,25]. However, the level of cell density that they achieved was lower compared to the use of spheroids. Previous studies also showed the interaction between ASC and MSC spheroids with hydrogels and polymeric scaffolds [13,26,27,28,29]. However, these spheroids remained as single spheroids throughout the period of culture. In the study developed by Shanbhag and collaborators (2021) [29], it is possible to observe that, although the bmMSC spheroids show a high cell viability in the 3D-printed polymeric scaffolds, the spheroids showed a low adhesion on their surface. The pore size of polymeric scaffolds can influence the behavior and differentiation potential of stem/stromal cells [30,31,32]. Here, we used 3D printing technique to fabricate the PLA/CHA scaffolds to achieve a higher reproducibility in terms of the pores and overall scaffolds sizes. 3D printing allows the deposition of cells, growth factors, and biomaterials layer by layer in high resolution [33,34]. The ASC spheroids had an average size of 400 µm, and the pore sizes of the PLA/CHA scaffolds had an average of 500 µm. In addition, the scaffolds had a diameter of 8 mm to match the size of the rat critical-size calvaria defect model [35]. 

The secretory capacity of soluble mediators with the ASC spheroidal constructs was evaluated, and collectively, the levels of the pro-inflammatory cytokines, i.e., GM-CSF [36], IFNy [37], IL-15 [38], and IL-12p70 [39], showed a low secretion with ASC spheroidal constructs at 1 week of culture. In addition, the secretory levels of IL-8 and VEGF, described as key soluble mediators that stimulate angiogenesis during bone development [40,41,42], were high for ASC spheroidal constructs at 1 week of culture. In addition, it was observed that a high secretion of IL-6 played a relevant role during osteogenesis [43]. Few studies have explored the inflammatory profile of ASC or bmMSC spheroids for tissue engineering applications. Souza and collaborators (2019) [44] showed a low amount of pro-inflammatory cytokines produced with SAOS-2 spheroids after 3 days of culture. Similarly, Shanbhag and collaborators (2020) [29] also observed a low amount of pro-inflammatory cytokines with bmMSC spheroids after 2 days of culture. 

Other studies showed that constructs based on hydrogels or printed polymers, aiming at bone tissue engineering strategies, can successfully release a high quantity of VEGF and anti-inflammatory cytokines in vitro [45,46,47]. However, the number of studies that explored the functional response of spheroids seeded in scaffolds is still scarce. Maia-Pinto and collaborators (2021) [28] produced osteoblast spheroids that could be seeded on PLA scaffolds. When the secretory activity was evaluated, it was possible to observe the secretion of VEGF, PDGF, and FGF. However, the levels of VEGF were 10 times lesser when compared to those observed in this study. 

Following the implantation, the constructs were analyzed after 1, 3, and 6 months for inflammatory response and connective tissue and new bone tissue formation at the defect area. So far, few studies have been published that investigate bone regeneration using spheroids [7,12,29,48] and 3D-printed scaffolds seeded with spheroids [12,29]. 

Previously, Lee and collaborators (2020) [12] explored the regenerative capacity of calvaria defects with 3D-printed micro-chambers containing ASC spheroids. The authors also observed that the group containing the ASC spheroids resulted in a larger regenerated bone area. Shanbhag and collaborators (2021) [29] explored the regeneration capacity of calvaria defects of rats with MSC spheroids encapsulated in 3D-printed polymeric scaffolds. As the main result, the authors observed bone formation in the groups containing the spheroids. Yanagi and collaborators (2021) [48] showed that dedifferentiated fat cell spheroids were also able to form new bone tissue in calvaria defects of rats. Similarly, Yamada and collaborators (2022) [16] explored the capacity of ASC spheroids only to regenerate calvaria defects. The authors observed a significantly higher regeneration capacity when compared to that of the control group. However, these authors did not explore a synergic strategy for bone regeneration. In addition, the bone formation was evaluated up to 3 months after implantation, while in this study, the follow-up was performed for up to 6 months to better investigate mature bone formation. 

In addition, previous studies showed the potential of PLA scaffolds to promote bone regeneration in critical-size models of rats [49,50,51]. Accordingly, we also observed bone formation in the PLA/CHA only group at 3 and 6 months, showing that the scaffold alone is indeed osteoinductive and can be successfully used in bone tissue engineering. Based on previous studies, the PLA/CHA’s osteoinductive capacity can be attributed to the presence of CHA [52]. More importantly, the use of ASC spheroids in a synergic strategy revealed similar results, including the absence of inflammatory response, suggesting a regenerative potential of this strategy for bone critical-size defects. However, in a future study, biofabrication approaches such as 3D bioprinting can be applied to achieve a higher homogeneity for the ASC spheroid distribution on the surface of the PLA/CHA scaffold.

In summary, a set of results suggests that ASC spheroidal constructs hold the potential to promote bone regeneration in critical-size calvarial defects due to the following reasons [1]: a high adhesion and interaction with most areas of the 3D-printed PLA/CHA scaffold in vitro; [2] a high secretion of VEGF, IL-6, and IL-8, which suggests a strong osteogenic and angiogenic potential; [3] a low secretion of proinflammatory soluble mediator factors as GM-CSF, IFNy, IL-15, and IL-12p70; and [4] a higher quantity of new bone tissue together with a lower quantity of connective tissue formed after 6 months. To optimize results in the presence of ASC spheroids, we hypothesize that two critical factors need to be improved: [1] the seeding of ASC spheroids using 3D bioprinting to increase the homogeneous distribution of spheroids on scaffolds; [2] and the use of polymers that show a higher biodegradability capacity compared with PLA. 

## 5. Conclusions

Therefore, the synergic strategy explored in this study, combining ASC spheroids and the 3D-printed PLA/CHA scaffolds, allowed a high cell density, due to the successful adhesion of the spheroids on the surface of the scaffold, together with a high secretion of VEGF, IL-6, and IL-8. Both the PLA/CHA and ASC spheroidal constructs were able to form new bone tissue in vivo in a critical-size defect rat model. Future studies using 3D bioprinting approaches are necessary to optimize the seeding of the ASC spheroids on the surface of PLA/CHA scaffolds, leading to a more homogeneous organization of the constructs and better in vivo regenerative responses. In addition, the use of PCL scaffolds seeded with ASC spheroids can optimize the bone formation in vivo as they have higher biodegradability properties. 

## Data Availability

Data is contained within the article.
